# Were our Ancestors Right in Using Flax Dressings? Research on the Properties of Flax Fibre and Its Usefulness in Wound Healing

**DOI:** 10.1155/2020/1682317

**Published:** 2020-11-24

**Authors:** Tomasz Gębarowski, Benita Wiatrak, Maciej Janeczek, Magdalena Żuk, Patrycja Pistor, Kazimierz Gąsiorowski

**Affiliations:** ^1^Department of Basic Medical Sciences, Wroclaw Medical University, Borowska 211, 50-560 Wroclaw, Poland; ^2^Department of Pharmacology, Wroclaw Medical University, Mikulicza-Radeckiego 2, 50-345 Wrocław, Poland; ^3^Department of Biostructure and Animal Physiology, Wroclaw University of Environmental and Life Sciences, Kożuchowska 1/3, 51-631 Wroclaw, Poland; ^4^Department of Genetic Biochemistry, University of Wroclaw, Przybyszewskiego 63/77, 51-148 Wroclaw, Poland; ^5^Faculty of Veterinary Medicine, Wroclaw University of Environmental and Life Sciences, Kożuchowska 1/3, 51-631 Wroclaw, Poland

## Abstract

**Background:**

Despite the wide range of medical dressings available commercially, there is still a search for better biomaterials for use in the treatment of especially difficult-to-heal wounds. For several years, attention has been paid to the use of substances, compounds, and even whole plants in medicine. Flax is a plant that has been used as a dressing for thousands of years. Therefore, we decided to test flax fibres that had previously been genetically modified as a potential wound dressing.

**Materials and Methods:**

In this study, two modified flax fibres and their combinations were tested on cell lines (mice fibroblast, normal human dermal fibroblast, normal human epidermal keratinocytes, human dermal microvascular endothelial cell, epidermal carcinoma cancer cells, monocyte cells). In the tests, fibres of the traditional flax (Nike) were used as a control. Several experiments were performed to assess cell proliferation and viability, the number of apoptotic cells, the cell cycle, genotoxicity, the level of free oxygen radicals, and determination of the number of cells after 48 hours of incubation of cell cultures with the tested flax fibres.

**Results:**

The obtained results confirm the positive influence of flax on the used cell lines. Both traditional fibres (Nike) and genetically modified fibres increased the proliferation of fibroblast cells and keratinocytes, reduced the level of free oxygen radicals, and influenced the repair of DNA damage. At the same time, the tested flax fibres did not have a proproliferative effect on the neoplastic cell line. Interestingly, genetic modifications had a stronger impact on the proliferative activity of fibroblasts, keratinocytes, and microvascular endothelium compared to the traditional flax fibre used.

**Conclusions:**

In this study, the positive properties of the tested flax fibres on cell lines were proved. In the next stage, it is worth carrying out *in vivo* tests of tested genetically modified flax fibres.

## 1. Introduction

It is well known that linen was used in medicine in ancient times. It was used, among others, to dress wounds and stabilize fractures [1]. Wound care was important, for example, due to frequent armed conflicts. In Mesopotamia, linen was used as bandages and tampons. However, wool material was more popular [1]. Cotton wool, made from the cotton tree, was known from the rules of Assyrian king Sennacherib [1]. Some texts give detailed instructions for surgery with a scalpel, including postoperative care such as the dressing of operations sites with oil-soaked linen bandages [2]. AMT 16/5 text describes the support of an infected purulent wound using honey-soaked linen compresses [3].

In Ancient Egypt, linen was the basic dressing material used in various forms. For example, it is mentioned in medical Edwin Smyth Papyrus: “…thou shouldst make for him two swabs of linen, (and) thou shouldst clean out every worm of blood which has coagulated on the inside of his nostril” [4]. It seems to be that treatment with linen, oil, and honey was considered standard wound dressing for soft tissue injuries in Ancient Egypt as it is recommended in 30 out of the 48 cases in the Edwin Smith Papyrus. Linen was used for its absorptive properties, which is useful in drawing moisture and lymph away from the wound [4]. Similarly to Mesopotamia, it was also used to stabilize fractures. In Edwin Smith Papyrus, various kinds of splints with linen are described: (a) brace of wood padded with linen (Edwin Smith, case 7) inserted into the mouth to help in feeding the patient, (b) splint made of linen (Edwin Smith, case 35, fractured clavicle), (c) stiff post-like roll of linen (Edwin Smith, cases 11 and 12), and (d) it is possible that cartonnage was used, similar to our plaster of Paris to splint fractures, also made of linen [5].

The oldest information on the use of bandages in ancient Greece comes from Homer's Iliad.

Homer's *Iliad* and *Odyssey* are our first sources of information about trauma management in the Greek (and Western) world. This poet recorded some 147 wounds, of which 106 were caused by spears, 17 by swords, 12 by arrows, and another 12 by slings [6]. In *Corpus Hippocraticum*, the linen bandages are recommended, among others, for the stabilization of the fractured humerus [7]. As a result of Alexander the Great's conquests, Greek medicine has taken over and established many Middle Eastern treatments. Alexandria in Egypt became the center of world science including medicine.

The Roman Republic and later the Empire waged wars on an unprecedented scale.

The Roman army had professional medical and veterinary staff in each legion. On the other hand, the gladiator fights have also gained valuable experience in the treatment of injuries and wounds. In the first step of proceeding, a medicus cleansed the wound with rainwater or other freshwater mixed with ammoniacum. Then, if it was possible, the wound sutured the wound with silk, linen, or gut thread, and whenever possible, the wound was closed [8]. The linen bandages were the most common type of dressing used for binding wounds. However, Cornelius Celsus recommended covering the wound with a special mixture that contains copper acetate, lead oxide, alum, dried pitch, dried pine-resin, oil, and vinegar [8]. Cornelius Celsus and Claudius Galen advised using plasters and linen bandages in the treatment of limb ulcers [9]. In the Middle Ages, the same means were still used, as in antiquity, although surgeons were no longer medicine doctors.

A revolution in synthetic products took place in the 20th century. By the 1950s, the textile industry produced synthetic fibres and fabrics from polymers, including nylon, polyethylene, polypropylene, polyesters, polyvinyl, acrylics, and olefins [10]. In medical applications, linen has been replaced by synthetic materials—relatively cheap and easy to manufacture (with the current technology). Synthetics have generally replaced natural materials in the last decades. Recently, however, there has been a trend towards a return to the use of natural materials in medicine. There are two companies in Poland that produce flax dressings, but they are still niche products on the market. Therefore, we drew attention to linen, which has been used as a dressing material since antiquity. Moreover, we currently have the possibility of influencing the properties of flax fibres by genetic modifications.

The study is aimed at investigating the effect of fibre from traditional cultivar of flax and fibres from two transgenic flax plants on cell cultures, commonly used as *in vitro* models of wound healing. The research results show that flax fibres have valuable properties in this field.

## 2. Materials and Methods

### 2.1. Tested Flax Fibres

The study examined the fibre obtained from a traditional fibrous cultivar of flax (Nike) and the fibres from two transgenic flax types (M50 and B14), and a combination of these two flax fibres (M50+B14) [11]. Both transgenic plants are obtained from Nike cultivar. B14 plants transformed with a defence-related potato *β*-1,3-glucanase gene (PR-2). M50 plants have been enriched with Ralstonia eutropha genes coding for *β*-ketothiolase (phbA), acetoacetyl-CoA reductase (phbB), and PHB synthase (phbC) for poly-*β*-hydroxybutyrate (PHB) synthesis.

Flax fibres were prepared for cell culture studies by dividing the fibres into four different weights: 10, 20, 30, and 40 mg. The fibres prepared in this way were soaked in PBS for 48 h, sterilized for 60 min by irradiation with UV, and transferred to cell cultures for 48 h. The fibres were in direct contact with the cells during the experiments.

### 2.2. Cell Lines and Conditions

This study used three human cell lines that are an *in vitro* skin model and one line of mouse fibroblasts recommended by the National Institutes of Health (NIH) for a cytotoxic assay. Research has also been carried out on a cell line often used to study monocyte and macrophage differentiation, including inflammation.

Normal human dermal fibroblast (NHDF) was purchased from Lonza (Verviers, Belgium) and were grown in Dulbecco's modified Eagle medium (DMEM) without phenol red. Normal human epidermal keratinocytes (NHEK) were from PromoCell (Heidelberg, Germany) and cultured in the KBM-Gold medium (Keratinocyte Cell Basal Medium). Human Dermal Microvascular Endothelial Cells—Adult (HMVEC) were purchased from Lonza and were cultured in EGM medium, supplemented according to Lonza's procedure. Cancer cells A431, which are derived from epidermal carcinoma, received from ATCC (USA) were incubated in Dulbecco's modified Eagle's medium (DMEM). Mice fibroblast BALB/3T3 (cloneA31 from mouse embryo donor) were obtained from Sigma-Aldrich (St.Louis, MO, USA) and were cultured in Minimum Essential Medium (MEM). Monocyte cell line (THP-1), which was derived from acute monocytic leukemia, was in receipt of the Laboratory for Therapeutic Innovation, Faculty of Pharmacy, University of Strasbourg, France. These cells were grown in suspended in RPMI-1640 medium. All cell lines were cultured in 95% humidified, at 37°C and 5% CO_2_ incubator, in the complete culture medium. The MEM, DMEM, RPMI-1640, and DMEM without phenol red media were supplemented with penicillin (10000 U/ml), streptomycin (10mg/ml), L-glutamine (200mM), and 10% fetal bovine serum (FBS). The KGM medium was supplemented following the PromoCell company's recommendations.

The cells were evaluated at least twice a week under a microscope, and then, the cells were passaged using the TrypLE solution or the appropriate medium was changed. All assays were performed in five independent replicates.

### 2.3. Cell Viability

The impact of flax fibres on the viability of the Balb/3T3 cell line was evaluated. After 24-hour treatment of cells with the tested fibres, the fibres were rinsed with PBS, which was collected to preprepared centrifuge tubes. The culture supernatant was collected into the same tubes. The culture plates were washed with TrypLe solution, which was also harvested. The TrypLe solution was again added, and culture plates were incubated for 2min at 37°C. Finally, the solution with cells was collected into appropriate tubes, which was centrifuged at 600 g for 5min. After supernatant removal, the cells were resuspended in PBS, and propidium iodide was added. After 5min incubation in the dark, the samples were analyzed in the image-based cytometer Arthur (NanoEnTek Inc.).

### 2.4. Cell Proliferation

The sulforhodamine-B (SRB) assay was used to evaluate the effect of flax fibres on the total amount of cellular protein. All cell lines were seeded on 96-well plates at a density of 2 × 10^4^ cell/ml per well. After 24-hour incubation, the tested fibres (with various weights: 10-40 mg) were put on cells for 48 h. Before the addition of the fibres, one plate from each cell line was fixed with cold 50% trichloroacetic acid (TCA) for the adherent cell line and 20% TCA for THP-1 cells at 4-8°C; these plates were the control. After 48-hour incubation, the culture plates were also fixed with 50% TCA for adherent cell line and 20% TCA for THP-1 cells for 1h at 4-8°C. In the next step, the plates were washed five times under running water. After air-drying, the sulforhodamine-B dye was added for a further 30 minutes to stained cellular proteins, and unbounded dye was removed by five washes with 1% acetic acid and again air-dried. In the last step, the SRB dye was dissolved with Trisma, and the absorbance was measured at 555nm, using a microplate reader (Victor2, PerkinElmer).

### 2.5. Evaluation of the Intracellular Free Radical Level

The intracellular reactive oxygen species (ROS) level was visualized by 2', 7'-dichlorofluorescein-diacetate (DCF-DA, BRAND) florescence. This assay was performed only for the NHDF cell line. These cells were seeded on 96-well plates at a density of 2.5 × 10^4^ cell/ml per well. The next day, into the plates, there were put tested flax fibres for 24h incubation. The supernatant and fibre were then removed, and the DCF-DA solution was added to the culture for the last 1 hour of incubation. The following steps were performed in two different ways. In the first case, after incubation with DCF-DA solution, the fluorescence was measured (*λ*_ex._ = 485nm, *λ*_em._ = 535nm) using a microplate reader (Victor2, PerkinElmer). In the second stage, during incubation with DCF-DA solution, for the last 30min, the cells were treated with 100*μ*M H_2_O_2_. In both cases, after incubation with the DCF-DA solution, before measuring, the supernatant was removed and cells were washed twice with PBS.

### 2.6. Total Number of Cells

An image-based cytometer, Arthur (NanoEnTek Inc.), was used to assess the effect of the tested flax fibres on cell proliferation. After 48 hours of incubation of Balb/3T3 and NHDF cell lines with the tested fibres, the supernatant was collected into previously prepared tubes, and the tested fibres were washed in PBS, which was also harvested into tubes. Cell cultures were washed with a TrypLE solution, which was also collected, and the TrypLE solution was added to the cells, and the plates were incubated at 37°C for 2min. The cell suspensions were harvested into the same tubes, which was then centrifuged at 600 g for 5min. The supernatant was removed, and the pellet was resuspended in PBS. In the last step, the cell suspensions were analyzed using a cytometer.

### 2.7. Apoptotic and Necrotic Cells

The effect of flax fibres on apoptosis and necrosis of NHDF and THP-1 cells was assessed. After 24-hour incubation of the cells with the tested flax fibres, the fibres were washed with PBS, and the washing solutions were collected in appropriate preprepared centrifuge tubes. The culture supernatant was also received into the same tubes. In the case of the NHDF cell line, all wells were then treated with the TrypLE solution and incubated at 37°C for 2min. In the next step, the solution with cells was harvested to the centrifuge tubes. For both cell lines, the tubes with cells were centrifuged at 600 g for 5min. After the supernatant was removed, the cell pellet was resuspended in 100*μ*l of HEPES–NaOH buffer at pH 7.5. The mixture of fluorochromes Annexin V-FITC and propidium iodide was then added and left in the dark for 10 min. The samples were analyzed in the image-based cytometer Arthur (NanoEnTek Inc.).

### 2.8. Cell Cycle

The effect of the flax fibres on the cell cycle of NHDF cells after 24-hour incubation was evaluated. After the incubation of cells with the tested fibres, a standard procedure was performed (such as detached cells from the surface of wells, centrifuged, and removed supernatant). The cells were then fixed with 70% cold ethanol for 10min at room temperature and again centrifuged at 600 g for 5min. The supernatant was removed, and the cell pellet was resuspended in propidium iodide solution and left in the dark for 10min. The samples were analyzed in the image-based cytometer Arthur (NanoEnTek Inc.).

### 2.9. Genotoxicity Assessment

The comet assay was performed to assess the effect of flax fibres on genotoxic damage and reparation after exposure to exogenic oxidative stress. After 48-hour incubation of NHDF cells with the tested flax fabrics, the cells were detached from the surface of the wells with the TrypLE solution, and they were collected in preprepared appropriate tubes and were added a medium with the serum to neutralize the effect of TrypLE. The tubes were centrifuged at 600 g for 5min. At this stage, DNA damage was carried out according to the standard comet test procedure, or to assess the repair activity of the tested fibres after supernatant removal, the cell pellet was resuspended with 200*μ*M H_2_O_2_ in PBS for 30min at 4°C, and the cell suspension prepared in this way was subjected to the standard comet test procedure. In both cases, after slides were stained with 1*μ*g/ml DAPI for 20 min, the samples were evaluated under a fluorescence microscope (Nikon Eclipse E-600) with UV 1A filter block and with a digital camera and CometPlus 2.5 software (ThetaSystem Electronics Gmbh, Gröbenzell, Germany). The 75 photos of the first comets found on each slide were taken. Using the software, the DNA content in the comet's head (%) and comet's tail length were analyzed.

### 2.10. Statistical Analysis

All studies were performed in five independent replicates. Due to the normal distribution and equal variance of the obtained results, statistical calculations were performed with parametric tests. Using the Statistica v.13 software, statistical significance was calculated using Tukey's post hoc test. The significance point was set at ^∗^*p*<0.05.

## 3. Results

### 3.1. Morphological Characterization of the Flax Fibres Used in the Experiment

The types of flax analysed did not differ significantly in the content of the basic polymers of the cell wall (cellulose, pectins, lignin, hemicellulose). Their differentiating parameter was the content of potentially bioactive substances such as phenolic compounds, polyhydroxybutyrate/hydroxybutyrate, and polyamines (Table 1).

### 3.2. Cell Viability

**Figure 1:**
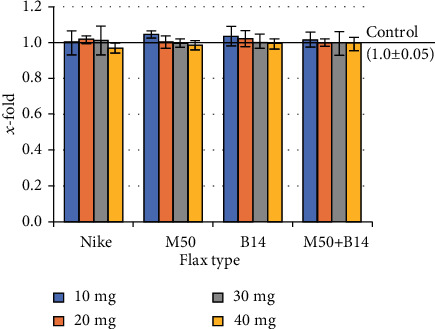
Cell viability of the Balb/3T3 cells after 48-hour incubation with the tested flax fibre in four different weights (10, 20, 30, and 40mg). The results are presented as *x*-fold–ratio to control culture (cells without fibres; *x* − fold = 1.0). The results are the means of 5 independent experiments ± SEM. The statistical significance of the differences between the results for the tested flax fibres compared to the control was calculated using Tukey's post hoc test (^∗^*p*<0.05).

The Balb/3T3 cell line is recommended by the NIH for assessing the effect of substances on cytotoxicity. The cytotoxic effect of the studied fibres on mouse fibroblasts was performed by analyzing the number of necrotic cells after propidium iodide staining. All flax fibres tested did not increase the number of necrotic cells compared to the control cultures (incubated only in complete medium) (Figure 1). The highest number of necrotic cells was observed for 20mg of traditional flax fibres (Nike), about 4% more than in the control culture. In other cases studied, the number of necrotic cells was not more than 1% compared to the control.

### 3.3. Cell Proliferation

**Figure 2:**
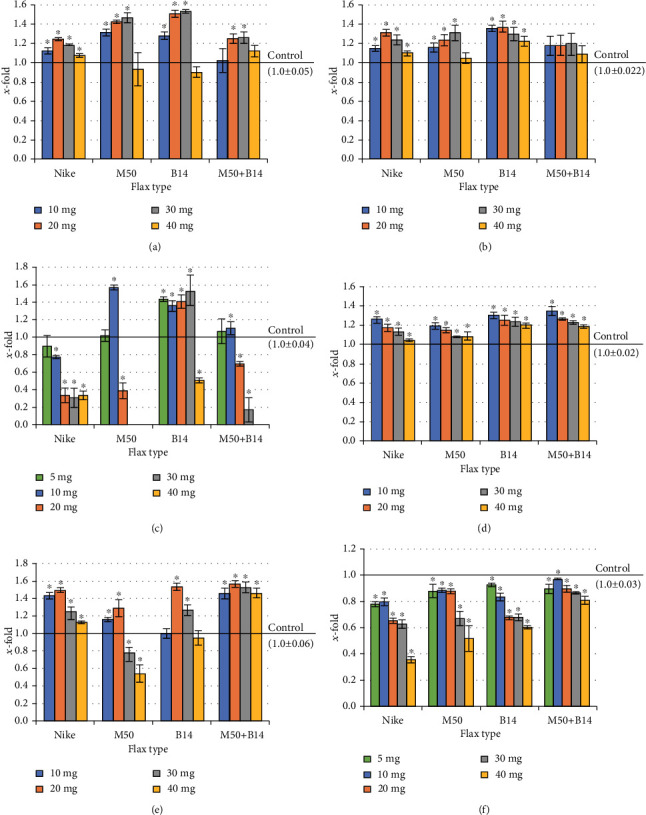
The proliferation of cells ((a) Balb/3T3, (b) NHDF, (c) THP-1, (d) NHEK, (e) HMVEK, (f) A431) after 48-hour incubation with the tested flax fibre in four different weights (10, 20, 30, and 40mg). The results are presented as *x*-fold–ratio to control culture (cells without fibres; *x* − fold = 1.0). The results are the means of 5 independent experiments ±SEM. Statistical significance of differences between the results for the tested flax fibres compared to the control was calculated using Tukey's post hoc test (^∗^𝑝 < 0.05). In THP-1 cells for weights of 30 and 40mg of the M50 flax fibre and 40 mg of M50+B14, there was complete inhibition of cell growth, and therefore, these results are not shown in the figure.

Flax fibres affected the amount of total cellular protein in the cultures of mouse fibroblasts (Balb/3T3), as well as human fibroblasts (NHDF) after incubation (48h). In the Balb/3T3 cells, no significant reduction in the protein level was found, regardless of the flax fibre used, which proves the lack of cytotoxicity (Figure 2(a)). At weights of 20-40 mg, an increase in total cellular protein was observed for all flax fibres compared to the control. The increase was found at all doses tested after using the traditional flax fibre (Nike) for both fibroblast lines, and B14 fibre in human dermal fibroblast culture (Figure 2(b)). In the 10-40mg concentration range, the increase in total Balb/3T3 cell protein was significant after incubation of the culture with M50 and B14 fibres (Figure 2(a)). In the same concentration range, the total growth of NHDF cell protein was observed after treatment with M50 fibre (Figure 2(b)). The combination of both fibres (M50+B14) resulted in an increase in the total amount of cellular protein in both fibroblast lines (Figures 2(a) and 2(b)). However, this increase was statistically significant only at doses of 10 and 30mg for mouse fibroblast culture (Figure 2(a)).

The first phase of wound healing preceding the proliferative phase is the inflammatory phase. Wound healing consists of three overlapping healing phases: inflammation, proliferation, and remodeling. In the inflammatory phase, there is an increase in the number of cytokines and immune cells that allow the wound to be cleansed. Inflammation should disappear in subsequent stages of the regeneration of the damaged skin. The model line in assessing inflammation is the THP-1 line. Therefore, the effect of the tested fibres on the total amount of THP-1 cell protein after 48 hours of incubation was evaluated. Nike fibre inhibits the total cellular protein over the entire range of concentrations tested (also, 10-40mg statistically significant). An activity increasing the amount of cellular protein, M50 fibre, and a combination of M50 and B14 fibres at a dose of 10mg (statistically significant), at the lowest concentration, this activity was comparable to the control culture (Figure 2(c)).

In comparison, at 20mg (M50 fibre) and 20-30mg (M50+B14 fibres), a decrease in the amount of cell protein was observed compared to control. Interestingly, at concentrations of 30-40 mg for the M50 fibre and the highest concentration of M50 and B14 combination fibres tested, the observed reduction in the amount of cellular protein compared to cell cultures before fibre addition indicates the cytotoxic effects of these fibres at high doses, which is important in the later stages of wound healing. The 48-hour incubation of THP-1 culture with B14 fibre strongly influences the increase in total protein in the concentration range of 5-30 mg. However, at the highest concentration tested, it has an inhibitory effect on cellular protein amount compared to the control (Figure 2(c)).

In wound healing, in the proliferation phase, an increase in the number of keratinocytes is the most important. Therefore, the effect of the flax fibres on the total amount of cellular protein in the culture of human keratinocytes was evaluated (Figure 2(d)). A statistically significant increase in the total amount of protein was observed for all tested fibres in the whole tested concentration range compared to the control. A concentration dependence was observed for all tested fibres, where the largest increase in protein was shown at the lowest concentrations (5mg). The highest increase in protein was observed in cultures treated with fibre combination of both modified fibres (M50+B14) (Figure 2(d)).

A very important stage of the proliferative phase in wound healing is richly vascularized granulation tissue. An assessment of the influence of the studied fibres on the amount of cellular protein in HMVEC vascular cell culture was carried out. The highest increase in cellular protein was observed at 20mg in all fibres examined. The traditional flax fibre (Nike) affected the growth of the total cellular protein over the entire concentration range studied. The M50 fibre at low doses (5 and 10mg) increased the amount of cellular protein (statistically significant) (Figure 2(e)).

In comparison, in higher concentrations (20 and 40 mg), a decrease in the total amount of HMVEC protein was observed compared to the control. However, after using B14 fibre, an increase in total protein was found in doses of 10 mg and 20 mg, while in other doses used, a similar amount of cellular protein was shown as in the control culture. Interestingly, the largest increase in cellular protein was observed after combining both modified fibres (M50+B14), even compared to the cultures treated with Nike fibre (Figure 2(e)).

Epidermal neoplasms are one of the most common skin cancers. They constitute about 65–75% of all skin cancers (about 25% of all cancers). It is expected that the given dressing material will not increase the cell division of the tumour cells. Therefore, the influence of the tested materials on the amount of cellular protein in the culture of epidermal cancer cells was checked (A431). There was a significant inhibition of the total amount of cellular protein compared to the control cultures in all tested fibres and the whole range of the tested doses. The strongest effect was observed after 48 hours of culture incubation in the presence of the Nike fibre (Figure 2(f)).

### 3.4. Total Number of Cells

**Figure 3:**
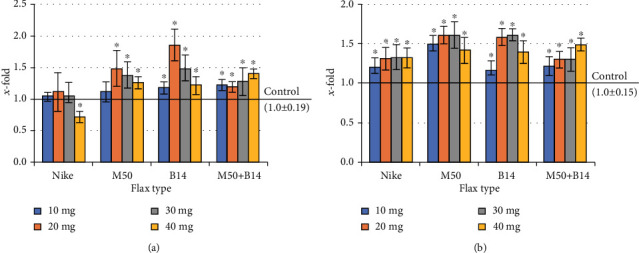
Total number of cells ((a) Balb/3T3, (b) NHDF) after 48-hour incubation with the tested flax fibres in four different weights (10, 20, 30, and 40mg). The results are presented as *x*-fold–ratio to control culture (cells without fibres; *x* − fold = 1.0). The results are the means of 5 independent experiments ± SEM. The statistical significance of differences between the results for the tested flax fibres compared to the control was calculated using Tukey's post hoc test (^∗^𝑝 < 0.05).

An increase in the total cellular protein may indicate an increase in cell mass or increased cell division (proliferation). Due to the expectation of increased skin fibroblast proliferation during intense wound healing, the number of NHDF and Balb/3T3 cells was evaluated after incubation for 48 hours in the presence of test fibres (Figure 3(a) and 3(b)).

After 48-hour incubation of Balb/3T3 cells with Nike fibre in the concentration range of 5-20 mg, a similar number of cells was observed as in the control. However, at the highest concentration tested, a reduced cell number was shown. In the tested M50, B14 fibres, and combinations of these two types of fibres, a significant increase in the number of Balb/3T3 cells was observed compared to the control (except for the 5mg dose after treatment with M50 fibre) (Figure 3(a)). In contrast, the number of human dermal fibroblast cells was higher compared to the controls at all doses and all tested flax fibres (Figure 3(b)).

### 3.5. Evaluation of the Intracellular Free Radical Level

**Figure 4:**
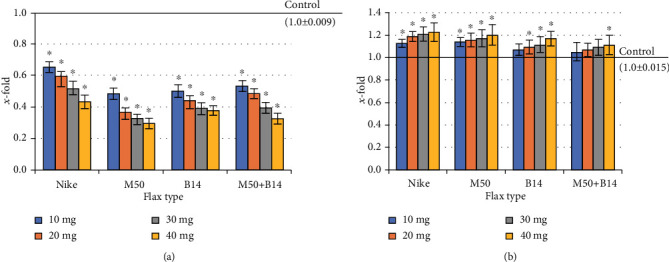
The level of ROS in NHDF cells cultured for 48 h with the presence of the tested flax fibres in four different weights (10, 20, 30, and 40mg): (a) subsequently exposed to H_2_O_2_ (100 *μ*M, 30 min, 4°C); (b) not exposed to H_2_O_2_. The results are presented as *x*-fold–ratio to control culture (cells without fibres; *x* − fold = 1.0). The results are the means of 5 independent experiments ± SEM. The statistical significance of differences between the results for the tested flax fibres compared to the control was calculated using Tukey's post hoc test (^∗^𝑝 < 0.05).

As the number of cells increases, the amount of free oxygen radicals increases as a result of metabolic processes through an increased number of cells. Therefore, the level of free radicals in the culture of NHDF cells treated with the tested fibres for 48 hours was evaluated. All the tested doses of all the tested fibres caused an increase in ROS levels of up to 20% compared to controls (except for the combination of M50 and B14 fibres, where the increase was significant only at the 40mg dose) (Figure 4(b)).

At the same time, a study was conducted to evaluate the effect of fibres on the ROS levels in cells that had previously exogenous oxidative stress by adding 100*μ*M H_2_O_2_. All the flax fibres tested reduced the level of free radicals compared to the control that was treated with 100 *μ*M H_2_O_2_ (Figure 4(a)).

### 3.6. Apoptotic and Necrotic Cells

**Figure 5:**
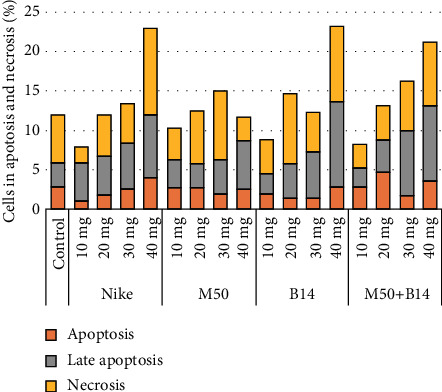
Apoptosis and necrosis of the NHDF cells after 48-hour incubation with the tested flax fibres in four different weights (10, 20, 30, and 40mg). The results are presented as the percentage of apoptotic and necrotic cells. The results are the means of 5 independent experiments.

To assess the number of cells in apoptosis and necrosis, Annexin V conjugated with FITC and propidium iodide staining was performed. After a 48-hour incubation of NHDF cells with the test fibres, the percentage of cells in necrosis did not exceed more than 5% (Figure 5). However, the sum of cells in the apoptosis and late apoptosis was the highest at the highest doses tested and did not exceed more than 22%. The lowest number of cells in the phase of apoptosis was observed in cultures treated with M50 fibre. Moreover, the sum of necrotic and apoptotic cells was not up to 10% (Figure 5).

### 3.7. Cell Cycle

**Figure 6:**
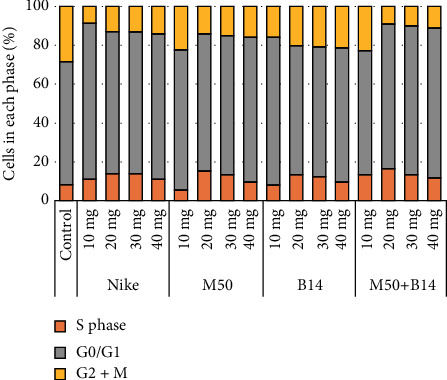
Cell cycle in NHDF cells after 48-hour incubation with the tested flax fibres in four different weights (10, 20, 30, and 40mg). The results are presented as the percentage of cells in each phase. The results are the means of 5 independent experiments.

A cell cycle analysis was performed to check whether incubation of the culture with the examined fibres increased the number of cells in the proliferation phase (S phase). After a 48-hour incubation of NHDF cells with the tested fibres, an increase in the number of S phase cells at 20-30 mg was observed in all cases (Figure 6).

### 3.8. Genotoxicity Assessment

**Figure 7:**
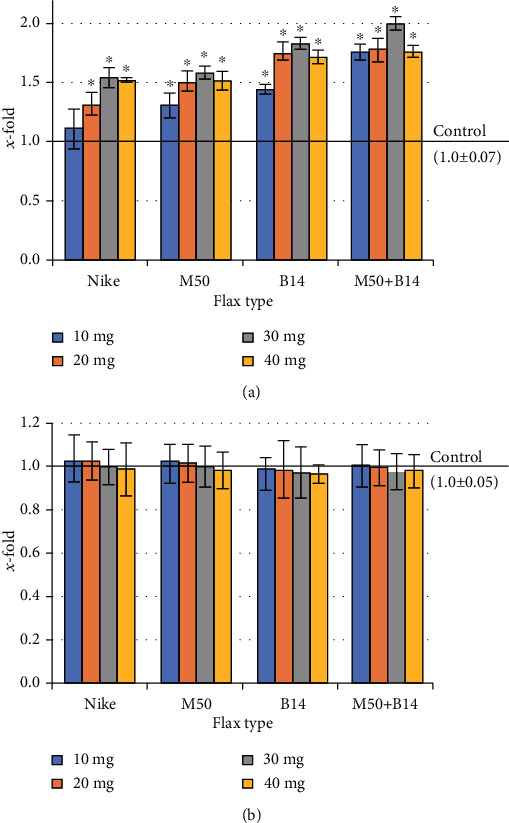
The level of DNA damage in NHDF cells cultured for 48 h with the presence of the tested flax fibres in four different weights (10, 20, 30, and 40mg): (a) subsequently exposed to H_2_O_2_ (100 *μ*M, 30 min, 4°C); (b) not exposed to H_2_O_2_. The results are presented as *x*-fold–ratio to control culture (cells without fibres; *x* − fold = 1.0). The results are the means of 5 independent experiments ± SEM. The statistical significance of differences between the results for the tested flax fibres compared to the control was calculated using Tukey's post hoc test (^∗^𝑝 < 0.05).

The effect on DNA damage in NHDF cells of the tested flax fibre was evaluated in the comet assay. The amount of DNA in the comet's head after incubation of NHDF cells with the tested fibres was compared to the amount of DNA in the control culture. No genotoxic effect has been demonstrated after applying all the tested fibres (Figure 7(b)). Therefore, the impact of the studied fibres on the repair of genotoxic damage caused by oxidative stress was checked. Oxidative stress was induced by incubation of cell cultures with H_2_O_2_. The amount of DNA in the head after incubation of cell cultures with the tested fibres was higher than in the control (culture incubated with H_2_O_2_) for all tested compounds (except for the lowest dose of the tested Nike fibre). The strongest reparations were observed after the incubation of cell cultures in the presence of combined M50 and B14 fibre at a dose of 30 mg (Figure 7(a)).

## 4. Discussion

Despite the wide range of various commercially available dressings, new solutions are still being sought in the treatment of particularly difficult to heal wounds. The flax has been used as a dressing for thousands of years. Its positive effect on injuries is well known [12–14]. Currently, it is used in the treatment of severely healing wounds, too [15, 16]. Flax is a plant that grows widely in the Middle East and temperate and Mediterranean climates. Cultivated for several thousand years, it serves as a source of oil and fibres for humans, not only in medicine but also in the textile, paper, cosmetics, and food industries. Flax fibre differs in composition from cotton fibre, and it contains mainly various polysaccharide polymers, such as cellulose (covers about 70% of fibre weight) and hemicellulose, the bundles of which are supplemented by pectin and phenolic polymer, lignin [17].

Wound healing is a multistage process consisting of three phases: (1) inflammation, cell proliferation, and tissue remodeling to restore tissue integrity after an earlier disruption of its continuity. Due to the rapid development of the transformation of modern molecular biology, research has been carried out for several decades to improve the quality of plant/fibre production [18] to develop the best-modified plants for use in medicine, including wound treatment. It enhances the productivity and quality of flax by influencing its length, thickness, softness, and elasticity. Genetic engineering allows us to identify and modify flax's bioactive components responsible for health-promoting properties. These changes should affect the immune cells that migrate to the lesions in the first phase. The purpose of these cells is to remove damaged tissue and initiate blood clotting processes. In our work, we showed a significant effect of the studied fibres on the increase in the amount of total protein of THP-1 cells (inflammation model) at low doses of M50 fibre, which indicates the possibility of using this fibre in the first phase of healing.

On the other hand, the combined M50+B14 fibre is recommended in subsequent phases because it was toxic to the model cell line in inflammation. In the second phase of wound healing, however, there is an intensive proliferation of cells along with the secretion of substances contained in the extracellular matrix (ECM). In the first stage of this phase, the formed tissue is highly vascularized and hydrated. At this stage, it is important to reduce inflammation, as shown by the use of higher concentrations of M50 and the combination of test fibres (M50+B14). Simultaneously, we showed an intense increase in cell proliferation—skin and endothelial fibroblasts after applying the tested fibres.

The metabolic profile of transgenic flax relates to changes in the level of, e.g., polyunsaturated fatty acids, lignan complexes, carotenoids, flavonoids, and phenylpropanoids [19–22]. In response to stress caused by the attack of pathogens (viruses, bacteria), the synthesis of lignin increases, which affects the cross-linking process and reduces the plasticity of the fibres. In transgenic flax fibres, there was an increase in the level of cellulose to lignin by silencing the CAD protein gene, an enzyme that catalyzes the biosynthesis of lignin monomers [23]. In the process of wound healing, it is very important to maintain a balance in the production of free radicals, both oxygen and nitrate. This disorder may affect the protracted phase of inflammation. Hence, the modifications mentioned above influencing the growth of flavonoids, which have anti-inflammatory and antioxidative activities, positively influenced the level of free oxygen radicals in our study. We have shown that during oxidative stress (caused exogenously by the presence of H_2_O_2_), a decrease in the level of free oxygen radicals was observed. At the same time, along with the reduction in the level of free oxygen radicals, increased repair of DNA damage was observed.

Preclinical studies of dressings from genetically modified flax were carried out in which the presence of a new metabolite of flax, which belongs to the terpenoids pathway, was observed. There are biological endocannabinoids (produced by human cells, e.g., anandamide or plant-derived terpenophenolics) and synthetic cannabinoids (e.g., HU-243, WIN-55,212-2). Reports show the important role of terpenophenol as an agent with analgesic and anti-inflammatory properties [24, 25]. Preclinical studies have demonstrated the antioxidant, anti-inflammatory, antiviral, and vasodilating properties of linseed dressings. The positive effects of treatment of difficult-to-heal wounds with new linen dressings characterized by greater hygroscopic properties and overproduction of polyphenols were manifested by reducing fibrosis, wound exudate, reducing the level of pain, promoting granulation, creating an effective barrier against unwanted tissues, fibrous tissue, and microorganisms [16].

Our previous studies of W92 fibres confirmed their positive effect on ulcer healing. The study applied the dressing for four weeks and then assessed the size of the wound and the exudate. It was shown that W92 fibres reduce exudate and wound size by 55% [26]. Based on these data, an attempt was made to modify flax to obtain better properties genetically. In this study, M flax is characterized by a genetic modification that influences a strong and stable overexpression of the cDNA. As a result, it affects the increased synthesis of polyhydroxybutyrate (PHD), *β*-ketothiolase (*phb* A), acetoacetyl-CoA reductase (*phb* B), and PHB synthase (*phb* C) (16). As a consequence of this modification, an improvement in the biomechanical properties of the new fibre was obtained by binding PHB with cellulose into polymers through hydrogen and ester bonds during plant growth. On contact with body fluid, PHB decomposes, releasing D, L-*β*-hydroxybutyrate, and as a normal component of blood and tissues, it promotes cell proliferation, preventing apoptotic cell death [27].

In conclusion, a limitation of this manuscript is the lack of assessment of the level of proinflammatory cytokines important in the wound healing process. Despite this, our study showed *in vitro* that modified flax possesses even better properties than the traditional flax. The investigation has shown clearly the beneficial effects of flax understudy on cell lines. It was shown that the level of necrosis was higher for conventional flax than modified flax. A higher level of total cell protein (fibroblast, keratinocytes, and dermal microvascular endothelial cells) concentration was observed for all flax types tested compared to controls. M50 and B14 showed high levels of reparations in the genotoxicity study turn; the lowest level of apoptotic cells was observed in cultures treated with the M50 fibre. The conducted tests showed that the tested, modified flax varieties have a positive effect on tissue cultures. The flax lines tested appear to be promising for further *in vivo* testing.

## Figures and Tables

**Table 1:** Content of potentially bioactive substances in tested flax fibres.

Bioactive substance	Content (ng/g) in flax fibres		
	Nike	B14	M50
Vanillin	156.34 ± 10.45	169.73 ± 11.83	147.49 ± 9.09
4-Hydroxybenzoic acid	344.50 ± 25.60	318.75 ± 32.46	354.48 ± 31.25
Ferulic acid	221.65 ± 21.65	245.61 ± 12.55	227.34 ± 9.83
Coumaric acid	62.23 ± 12.45	53.28 ± 10.33	68.42 ± 12.24
Syringaldehyde	148.17 ± 18.33	143.67 ± 12.24	172.74 ± 19.95
Polyhydroxybutyrate/hydroxybutyrate	14.67 ± 3.05	15.45 ± 4.02	32.40 ± 9.95
Polyamines	357.04 ± 19.95	417.87 ± 22.92	836.76 ± 28.25

## Data Availability

The data underlying this article will be shared on a reasonable request to Tomasz Gębarowski (email.: tomasz.gebarowski@umed.wro.pl).
